# Association between health insurance enrolment and maternal health care service utilization among women in Ethiopia

**DOI:** 10.1186/s12889-021-12105-9

**Published:** 2021-12-30

**Authors:** Abdu Seid, Mohammed Ahmed

**Affiliations:** 1 Department of Midwifery, Woldia University, Woldia, Ethiopia; 2 Department of Public Health, Woldia University, Woldia, Ethiopia

**Keywords:** Health insurance enrolment, Maternal health care, Ethiopia

## Abstract

**Background:**

Health insurance was considered as the third global health transition which can increase access to health care services by eliminating monetary obstacles to maternal health care use, particularly in emerging nations. Hence, this study aimed to assess the association between health insurance enrolment and maternal health care service utilization among women in Ethiopia.

**Methods:**

A cross-sectional study was conducted using the 2016 Ethiopia Demographic and Health Survey (EDHS) data set. About 4278 mothers who had delivered at least one child in the last five years of the survey were selected in the study. Multivariate logistic regression analysis was performed to measure the relationship between health insurance enrolment and maternal health care service utilization by controlling confounders An adjusted odds ratio with a 95% confidence interval and *p*-values < 0.05 were well-thought-out to state the imperative association.

**Results:**

The overall health insurance coverage among the women was 4.7%. About, 18.1% of women from households in the poorest wealth quantile had no health insurance coverage for maternal health care services. Moreover, 84% of women lived in a rural area did not enclose by health insurance. According to multivariate logistic regression, the likelihoods of ANC utilization were 1.54 times (AOR: 1.54; 95% CI: 1.06–2.25) higher among mothers who were enrolled in health insurance compared to their counterparts. In the same vein, the likelihoods of been attended by a skilled birth attendant were 1.84 times (AOR: 1.84; 95% CI: 1.1–3.08) higher among mothers who were enrolled in health insurance.

**Conclusions:**

This study has shown that women enrolled in health insurance were associated with skilled delivery and recommended ANC utilization than women who did not enroll in health insurance. Health insurance enrolment enterprises must be available to all pregnant women, particularly those of poorer socioeconomic rank.

## Background

To escalate access to maternal health care services in developing countries, health insurance has paramount importance through tackling financial barriers [1–3]. According to the World Health Organization’s (WHO) global agenda on Universal Health Coverage (UHC), all people should get comprehensive healthcare services at a reasonable cost and without monetary hardship by avoiding disastrous healthcare payment [4], which is a serious component of countrywide health policies in many low and middle-income nations [5]. Developing nations are experiencing reformation to expand their healthcare supporting mechanisms which able to increase the health care service utilization effectively and efficiently [6, 7]. Besides, the post-2015 Sustainable Development Goals (SDGs) have projected a new target to decrease the global Maternal mortality ratio (MMR) to 70 per 100,000 live births by 2030 [8, 9]. Likewise, the Ethiopian government launched a health sector development program for 2010–2015 which includes community-based health insurance (CBHI) for citizens and social health insurance for formal sectors to increase access to health care through risk pooling [10].

Recent evidence indicates that the higher frequency of antenatal care service utilization has a significant impact in reducing the probability of stillbirths, MMR, and perinatal deaths by eight per 1000 births as compared to four visits [11]. MMR in Ethiopia was 871 in 2000 EDHS, 676 in 2011 EDHS, and 412 in 2016 per 100,000 live births which is an insignificant change over the last decades [12]. Obstetrics complications like antepartum bleeding, postpartum bleeding, dystocia, preeclampsia, puerperal infection, and unsafe abortion were associated with maternal death in Ethiopia [13]. Additionally, delays in health-seeking behavior, accessing and receiving care for obstetric emergencies, financial, and health service-related factors were also mentioned as contributing factors to maternal death [13, 14]. The crude domestic product (GDP) per individual in Ethiopia become approximately US$360 with a growth rate of 7–8% in a year, and about 30% of its population surviving under the national poverty line, and a huge percentage of health costs funded via out-of-pocket many bills [15, 16]. Then, out-of-pocket expenses leftovers a chief supply of health financing for the Ethiopian population and it is a substantial obstacle for gain access to and using maternal health care services [17, 18].

Therefore, the implementation of health insurance enrolment can be the potential health bankrolling system in minimizing out-of-pocket fees, mainly in the place where numerous persons live in rural parts of Ethiopia [19].

Various studies showed that the effect of health insurance coverage on the usage of maternal healthcare services could validate the effective association among rural and women who had low income hence insurance leads to a 9–11% higher likelihood of using ANC, skilled delivery, and PNC utilization [20–22]. Likewise, a research finding done in Tanzania discovered that taking health insurance was positively associated with the recommended timing of ANC and skilled delivery practice [23]. Evidence from Gabon and Ghana showed that women’s enrolment in health insurance can increase the use of maternal healthcare [24, 25]. Besides, maternal healthcare use could be affected by health system factors, wealth index, residence, history of previous birth complications, number of children, transportation and, religion [26–31].

Unfortunately, no studies conducted in Ethiopia so far to measure the relationship between health insurance enrolment and maternal health care service utilization among Ethiopian women to know the extent of increasing maternal health care service utilization gained from insurance policies, which limits in reaching convincing results. So, this study is designed to measure the relationship between health insurance enrolment and utilization of maternal health care services in Ethiopia using a recent version of nationally representative data.

## Methods

### Data and sampling

This study was performed using national representative recorded data from the 2016 Ethiopia Demographic and Health Survey (EDHS). A full clarification of the data source, strategy, and methods of the survey become observed elsewhere [12]. This study used Woman’s data which was gained from all women aged 15–49 in carefully chosen houses. The women’s sample involves 15,683 women ages 15–49. Finally, weighting adjustment was done by considering the sampling procedure, Therefore, all women who delivered at least one child in the last five years (4278 women) before the survey were included.

### Study variables

The dependent variable of this study was maternal healthcare service utilization, which involved antenatal care (ANC), skilled birth, and postnatal care (PNC). ANC is the care given by skilled health professionals to gravid women and adolescent girls to safeguard the quality healthcare for both mom and fetus throughout her pregnancy [32]. Even though it is not practically applicable in Ethiopia, the new 2016 WHO guideline on ANC recommended that pregnant women should get eight contacts; which their first communication in the first 12 weeks’ gestation and next contacts should be done at 20, 26, 30, 34, 36, 38 and 40 weeks of pregnancy [11, 32]. Skilled delivery is having birth health institutions through qualified healthcare experts [33]. PNC is the care which given for the women by skilled healthcare expert after delivery [34]. Skilled delivery and postnatal care were coded ‘yes/no’ based on whether the women have gotten the service or not. Antenatal care was dichotomized based on WHO recommendations: those who had four or more antenatal visits were categorized as “complete recommended ANC” and others who had less than four as categorized as “not complete recommended ANC”. The principal exposure variable of this study was health insurance enrolment which dichotomized as (yes/no). Ethiopia has different types of health insurance which include social insurance, employer-based insurance, community-based insurance, and privately purchased commercial insurance. Those women enrolled in at least one of the types of health insurance for their last birth depicted above were considered as having health insurance coverage otherwise not.

Other independent variables have been carefully chosen from a literature assessment [26–31] on factors related to maternal health care service utilization which include; age, educational status, marital status, resident, wealth index, occupation, media access, distance to the health facility, pregnancy intention, and the number of offspring. The wealth index become labeled into five groups (the poorest, poorer, middle, richer and richest) which were found from 2016 EDHS.

### Data processing and analysis

Descriptive statistical findings and weighted proportions of socio-demographic data have been computed related to health insurance and maternal healthcare utilization. Rao scot chi-square analysis was carried out to pick contenders for the multivariate model. All covariates of *P*-values of < 0.25 had been entered all together into the multiple regression models. Adjusted odds ratios (AOR) had been used to state the significant relationship. The analysis had been done using SPSS version 24. Sample weighting has been done to manage the probabilities of sampling through strata and clusters.

In our study, the magnitude of the Variable inflation factor (VIF) was used to differentiate the multicollinearity issue. Even though no official standards exist for determining when a VIF is very huge, general cut-off values, such as VIF ≥5 or VIF ≥10; are usually used to govern [35]. In this study, the magnitudes of VIF for ANC, skilled delivery, and PNC were 3.5, 4.3, and 4.86 respectively; which shows that no multicollinearity issue is found.

## Results

### Maternal health insurance and related characteristics

About 4278 samples of women who delivered in the past 5 years preceding the survey were included in the study and analyzed. According to this finding, the overall health insurance coverage among women was 4.7%. According to this finding, health insurance coverage was quite different by their education level, occupation, wealth index, marital status, and place of residence. Maternal health insurance coverage decreased from non-educated women to secondary and above level (66.9 to 5.3%, *p* < 0.042). Likewise, 18.1% of women from households in the poorest wealth quintile had no health insurance coverage for maternal health care services. Moreover, 84% of women who are not covered by health insurance live in rural areas. Similarly, 91% of currently in a union or married women had no health insurance coverage. The likelihood of having health insurance was higher among those aged 25–34 years and among women who had ≥5 children. Additionally, more than half of (51.6%) of women who are not working were not under the health insurance enrolment scheme (Table 1).

**Table 1:** The distribution of respondents related to socio-demographic characteristics and health insurance coverage (*n* = 4278)

Variable	Categories	Maternal health insurance		Chi-square *p*-value
		Not insured (%)	Insured (%)	
Age	15–19	305 (6.60)	7(4.60)	0.006
	20–24	905 (20.90)	22 (12.0)	
	25–34	2425 (60.8)	134 (64.0)	
	35–49	444 (11.80)	36 (19.30	
Educational status	No education	2185 (57.30)	110 (66.9)	0.042
	Primary	1258 (31.50)	44 (22.60)	
	Secondary	416 (7.30)	21 (5.10)	
	Higher	220 (3.90)	24 (5.30)	
Marital status	Never in union	52 (1.2)	4(1.60)	0.009
	Currently in union	3653 (91.0)	184 (95.0)	
	Formerly in union	374 (7.80)	11 (3.40)	
Residence	Urban	1055 (16.0)	55 (16.70	0.86
	Rural	3024 (84.0)	144 (83.30)	
Wealth index	Poorest	1126 (18.10)	15 (6.0)	0.008
	Poorer	633 (19.8)	31 (18.60)	
	Middle	603 (21.40)	49(27.0)	
	Richer	541 (19.60)	40 (27.30)	
	Richest	1176 (21.0)	64 (21.10)	
Occupation	Not Working	2170 (51.60)	54 (31.20)	0.001
	Working	1909 (48.40)	145 (68.80)	
Media access	No	2446 (63.10)	96 (55.10)	0.097
	Yes	1633 (36.90)	103 (44.90)	
Distance	Big problem	2014 (54.40)	65 (38.60)	0.003
	Not a big problem	2065 (45.60)	134 (61.40)	
Pregnancy intension	Then	3272 (76.10)	150 (74.60)	0.868
	Later	537 (15.60)	32 (15.80)	
	No more	270 (8.40)	17 (9.60)	
Parity	One	1424 (31.60)	46 (20.10)	0.006
	2–4	1472 (36.60)	80 (39.80)	
	> = 5	1183 (31.80)	73 (40.10)	

### Maternal health care service utilization

Around 95, 95.2, and 94.7% of women who had no health insurance enrolment did not attend complete the recommended ANC visit, skilled delivery, and postnatal care service utilization respectively. Moreover, 66.7, 60.5%, and 24.8 mothers aged 25–34 years had attended the recommended ANC, skilled delivery, and PNC correspondingly. Similarly, 61.8, 71.8, and 58.9% of mothers who did not attend formal education did not receive ANC, skilled delivery, and PNC respectively. Likewise, 87.9, 96.7, and 84.8% of mothers residing in rural areas had not taken the recommended ANC visit, skilled delivery, and PNC respectively. Additionally, 67.4, 73.9, and 64.2% of women had no media access and did not receive the recommended number of ANC, skilled delivery, and postnatal care service correspondingly.

A higher proportion of women who in the richest quintile class received recommended ANC visit, skilled delivery, and PNC service (42.7,42.2, and 34.9%) respectively. Furthermore, 90.6, 90.2, and 90.7% of currently in a union or married women received recommended ANC, skilled delivery, and PNC service respectively.

In bivariate analysis, health insurance enrolment had a significant association with antenatal care, skilled delivery, and postnatal care service utilization (Table 2).

**Table 2:** Bivariate analysis to assess the relationship between the independent variables and maternal health care service utilization (*n* = 4278)

Variables	Recommended ANC		*P* value	Skilled Delivery		*P* value	Received PNC		*P*-value
	No (%)	Yes (%)		No (%)	Yes (%)		No (%)	Yes (%)	
**Health insurance**									
No	3121(95.0)	958(92.4)	0.001	2239 (95.2)	1840(93.4)	0.09	3773(94.7)	306(91.5)	0.06
Yes	126 (5.0)	73 (7.6)		76(4.8)	123(6.6)		169 (5.3)	30 (8.5)	
**Age**									
15–19	259 (6.9)	53 (4.6)	0.013	161 (6.0)	151(7.2)	0.000	297 (6.6)	15 (4.9)	0.45
20–24	716(20.5)	211(19.9)		437 (17.1)	490(25.7)		861(20.1)	66 (24.8)	
25–34	1870(59.6)	689(66.7)		1360 (61.2)	1199(60.5)		861(20.1)	66 (24.8)	
35–49	402(12.9)	78 (8.8)		357 (15.7)	123 (6.6)		2344(61.0)	215(59.4)	
**Education**									
No education	1962(61.8)	333(40.0)	0.000	1673 (71.8)	622(35.5)	0.000	2154(58.9)	141(43.5)	0.001
Primary	947(30.1)	355(35.3)		560 (26.1)	742 (38.9)		1197(30.7)	105(36.1)	
Secondary	240 (5.6)	197(14.1)		71 (1.7)	366 (15.7)		386 (6.8)	51 (12.0)	
Higher	98 (2.5)	146(10.6)		11 (0.4)	233 (9.8)		205 (3.7)	39 (8.3)	
**Marital status**									
Never in union	34 (0.9)	22 (2.3)	0.029	14 (0.7)	42(1.9)	0.041	50 (1.2)	6 (1.7)	0.74
Currently union	2921(91.3)	916(90.6)		2109(91.8)	1728(90.2)		3544(91.3)	293(90.7)	
Formerly union	292 (7.7)	93 (7.0)		192 (7.4)	193 (7.9)		348 (7.6)	37 (7.6)	
**Residence**									
Urban	565(12.1)	545(33.5)	0.000	143 (3.3)	967 (36.4)	0.000	984 (15.2)	126(27.6)	0.000
Rural	2682(87.9)	486(66.5)		2172(96.7)	996 (63.6)		2958(84.8)	210(72.4)	
**Wealth index**									
Poorest	1040(19.5)	101(8.5)	0.000	923 (23.8)	218 (7.5)	0.000	1098(18.2)	43 (6.9)	0.000
Poorer	569(21.6)	95 (11.7)		431 (22.8)	233 (14.9)		620 (20.4)	44 (11.6)	
Middle	539 (23.1)	113(15.5)		416 (24.5)	236 (17.1)		601 (21.7)	51 (21.0)	
Richer	455 (19.7)	126(21.6)		344 (21.1)	237 (18.3)		524 (19.6)	57 (25.6)	
Richest	644 (16.2)	596(42.7)		201 (7.7)	1039(42.2)		1099(20.0)	141(34.9)	
**Occupation**									
Not Working	1722(51.1)	502(47.7)	0.214	1263 (51.9)	961 (48.2)	0.133	2085(51.3)	139(38.3)	0.00
Working	1525(48.9)	529(52.3)		1052 (48.1)	1002(51.8)		1857(48.7)	197(61.7)	
**Media access**									
No	2197(67.4)	345(41.7)	0.000	1776 (73.9)	766 (44.9)	0.000	2398(64.2)	144(41.9)	0.000
Yes	1050(32.6)	686(58.3)		539 (26.1)	1197(55.1)		1544(35.8)	192(58.1)	
**Distance**									
Big problem	1726(56.5)	353(40.2)	0.000	1433 (62.0)	646 (40.0)	0.000	1963(54.8)	116(35.9)	0.000
No big problem	1521(43.5)	678(59.8)		882 (38.0)	1317(60.0)		1979(45.2)	220(64.1)	
**Pregnancy wanted**									
Then	2590(75.4)	832(78.4)	0.23	1844 (75.4)	1578(76.9)	0.18	3154(76.2)	268(72.8)	0.025
Later	419 (15.7)	150(15.1)		292 (15.2)	277 (16.2)		521 (15.1)	48(22.4)	
No more	238 (8.9)	49 (6.5)		179 (9.4)	108 (6.9)		267 (8.7)	20 (4.8)	
**Parity**									
One	1035(29.3)	435(38.6)	0.000	572 (21.8)	898 (45.6)	0.000	1356(30.9)	114(32.4)	0.003
2–4	1133(36.2)	419(39.4)		807 (36.5)	745 (37.3)		1417(36.1)	135(46.6)	
> =5	1079(34.5)	177(22.0)		936 (41.7)	320 (17.1)		1169(33.0)	87 (21.1)	

### Association between health insurance enrolment and maternal health care service utilization

All the variables were entered into multivariate logistic regression analysis. After adjusted for possible confounders by logistic regression, health insurance was positively associated with ANC and skilled delivery.

According to multivariate logistic regression, the odds of ANC utilization were 1.54 times (AOR: 1.54; 95% CI: 1.06–2.25) higher among mothers who were enrolled in health insurance. In the same vein, the odds of been attended by a skilled birth attendant were 1.84 times (AOR: 1.84; 95% CI: 1.1–3.08) higher among mothers who were enrolled in health insurance. Besides, mothers aged 25–34 compared to 15–19 years had approximately 2 times (AOR: 2.03; 95% CI: 1.15–3.58) higher odds of ANC service utilization.

Regarding educational status, the odds of ANC service utilization were 1.38 (AOR: 1.38; 95% CI: 1.05–1.81), 1.57(AOR: 1.57; 95% CI: 1.02–2.42), 2.1(AOR: 2.10; 95% CI: 1.27–3.42) times higher among women attended primary, secondary and higher education, respectively compared to non-educated one. Likewise, the odds of been attended by a skilled birth attendant were 1.79 (AOR: 1.79; 95% CI: 1.43–2.24), 4.84 (AOR: 4.84; 95% CI: 2.89–8.90), 6.82(AOR: 6.28; 95% CI: 3.18–14.6) times higher among women attended primary, secondary and higher education, respectively compared to non-educated one. Moreover, mothers residing in an urban area compared to rural residents had 4.74 (AOR: 4.74; 95% CI: (2.62–8.57) times higher odds of been attended by skilled birth attendants.

The odds of recommended ANC and PNC utilization were 1.45(AOR: 1.45; 95% CI: 1.08–1.95), and 1.67 times (AOR: 1.67; 95% CI: 1.05–2.64) higher among mothers who accessed media compared to its counterparts. The odds of been attended by a skilled birth attendant were lower by 51% (AOR: 0.49; 95% CI: 0.35–0.67) among mother who had 2–4 children compared to who had one child. Likewise, mothers who had children of 5 and above compared to mothers who had one child had a lower odds of ANC and skilled delivery by 41%(AOR: 0.59; 95% CI: 0.38–0.92), and by 64% (AOR: 0.36; 95% CI: 0.25–0.52), respectively (Table 3).

**Table 3:** Multivariate analysis to identify the relationship between health insurance and maternal health care service utilization (*n* = 4278)

Variables	Categories	Recommended ANC	Skilled delivery	PNC
		**AOR;95% CI**	**AOR;95% CI**	**AOR;95% CI**
Health insurance	No	Ref.	Ref.	Ref.
	Yes	1.54(1.06–2.25)*	1.84(1.1–3.08)*	1.32(0.7–2.46)
Age	15–19	Ref.	Ref.	Ref.
	20–24	1.31(0.77–2.21)	0.94(0.61–1.45)	1.33(0.59–3.03)
	25–34	2.03(1.15–3.58)*	1.02(0.63–1.64)	0.97(0.39–2.4)
	35–49	1.97(0.97–3.98)	0.74(0.41–1.32)	1.42(0.46–4.4)
Educational status	No education	Ref.	Ref.	Ref.
	Primary	1.38(1.046–1.81)*	1.79(1.43–2.24)*	1.21(0.75–1.94)
	Secondary	1.57(1.02–2.42)*	4.84(2.89–8.9)*	1.20(0.59–2.44)
	Higher	2.1(1.27–3.42)*	6.82(3.18–14.6)*	1.32(0.53–3.26)
Marital status	Never in union	Ref.	Ref.	Ref.
	Currently in union	0.42(0.17–1.00)	0.6(0.21–1.74)	0.75(0.23–2.47)
	Formerly in union	0.43(0.17–1.09)	0.58(0.19–1.72)	0.82(0.24–2.86)
Residence	Rural	Ref.	Ref.	Ref.
	Urban	0.96(0.61–1.5)	4.74(2.62–8.57)*	0.95(0.5–1.79)
Wealth index	Poorest	Ref.	Ref.	Ref.
	Poorer	1.15(0.72–1.84)	1.82(1.22–2.73)*	1.35(0.69–2.61)
	Middle	1.34(0.84–2.11)	2.03(1.39–2.96)*	2.18(1.13–4.19)*
	Richer	1.87(1.18–2.97)*	2.17(1.45–3.25)*	2.42(1.31–4.44)*
	Richest	3.02(1.79–5.10)*	3.71(2.17–6.35)*	2.19(1.07–4.45)*
Occupation	Not Working	Ref.	Ref.	Ref.
	Working	0.91(0.72–1.16)	1.03(0.82–1.30)	1.43(1.03–1.98)*
Media access	No	Ref.	Ref.	Ref.
	Yes	1.45(1.08–1.95)*	1.05(0.81–1.35)	1.67(1.05–2.64)*
Distance to a health facility	Big problem	Ref.	Ref.	Ref.
	Not a big problem	1.2(0.91–1.56)	1.26(0.97–1.63)	**1.6(1.08–2.36**)*
	No more	Ref.	Ref.	Ref.
Pregnancy intension	Then	1.25(0.79–1.97)	0.8(0.53–1.21)	1.67(0.83–3.37)
	Later	1.03(0.63–1.68)	0.62(0.37–1.05)	2.33(1.06–5.14)*
Parity	One	Ref.	Ref.	Ref.
	2–4	0.75(0.52–1.06)	0.49(0.35–0.67)*	1.48(0.85–2.57)
	> = 5	0.59(0.38–0.92)*	0.36(0.25–0.52)*	0.91(0.44–1.89)

*: shows a statistically significant association where *p* < 0.05; Ref.; Reference; Goodness of fit for ANC (Cox and Snell: 0.75; Nagelkerke: 0.81; McFadden: 0.78), Goodness of fit for Skilled delivery (Cox and Snell: 0.64; Nagelkerke: 0.85; McFadden; 0.83), Goodness of fit for Postnatal care (Cox and Snell: 0.78; Nagelkerke: 0.83; McFadden; 0.81)

Model Sensitivity analysis: ANC; 78.4%, skilled delivery; 75.5%, and Postnatal care; 92.1%.

## Discussion

In my understanding, this is the primary investigation to be performed in Ethiopia to validate the relationship between health insurance enrolment and maternal healthcare service utilization. The results of this study showed that maternal health insurance status had a substantial role in the utilization of ANC and skilled delivery. In contrast, health insurance has no significant association with PNC utilization.

According to this finding, the odds of ANC utilization were higher among mothers who had health insurance, which is consistent with a research accomplished in low and middle-income countries that the percentage of women having at least one ANC follow up was higher among women who had been covered with health insurance than their counterparts [36]. Likewise, this result is also reinforced by a study made in Gabon [24] and Ghana [30, 37, 38] in which women with health insurance are likely to have a high frequency of ANC follow-up. Generally, eliminating the economic obstacles to ANC is supposed to increase the use of ANC follow-up [21].

Regarding skilled delivery, the likelihoods of been attended by a skilled birth attendant were higher among mothers who were covered by health insurance. This result is steady with a study which has been done in Gabon [39], Nigeria [40], and across 3 countries (Ghana, Indonesia, and Rwanda) stated that health insurance possession significantly increased the chance of women having a birth in a health institution [20, 28, 37, 41, 42].

Similar to earlier research [43, 44], this study also revealed that obstetrics and sociodemographic factors which include; age, education level, wealth index, marital status, place of residence, distance to a health facility, media access, occupation, parity, and pregnancy intention were major forecasters of maternal healthcare service utilization. Specifically, this study also showed that the age of the mothers was one of the independent variables which affect the accomplishment rate of ANC follow-up. Mothers aged 25–34 compared to 15–19 had approximately higher probabilities of ANC service utilization. The result of this study is equivalent to earlier studies done in Cambodia [45], and Tanzania [23]. It is also supported by a study done in India in which adolescents had poor maternal healthcare utilization [46]. The consistency might be due to the older women could have bad obstetrics history in the previous pregnancy.

Similarly, women from the richest households are 3 times, more likely to use ANC and 3.7 times more likely to have skilled birth, and the likelihoods of PNC service were 2.19 times higher than poorest households which is analogous to a study done in Cambodia [45]. The similarity could be the fact that women from the wealthiest households could have learned, empowered, and get information about the complication of pregnancy, and therefore they follow continuum maternal healthcare service utilization.

Regarding educational status, the odds of ANC service and skilled delivery utilization were higher among women who attended primary, secondary, and higher education, respectively compared to non-educated ones. This finding is also in line with other studies done in Tanzania [23], and Uganda [47] Ethiopia [48].

Moreover, mothers living in an urban area had higher chances of been attended by a skilled birth attendant compared to rural residents, which are contradicted the study done in Tanzania [23] in which women of urban areas had lower odds of been attended by a skilled birth attendant. The discrepancy might be due to variation in socio-demographic factors.

Furthermore, the likelihoods of suggested ANC and PNC use were higher among mothers who accessed media compared to their counterparts, which is also reinforced by a study done in a developing country [43, 44]. Similarly, skilled delivery was lower among mothers who had 2–4 children than mothers who had one child. Likewise, mothers who had children of 5 and above compared to mothers who had one child had a lower odds of ANC and skilled delivery which is consistent with a study done in Cambodia [45], which implies women who had less order birth are significantly related with frequency of ANC follow up and their persistence to use skilled delivery. But this finding is contradicted with a study done in Tanzania [23] which stated women with grand para were more likely to have more information of predisposing factors and then they were followed to recommend continuum maternal healthcare.

Finally, this research revealed that pregnancy intention, occupation, and remoteness to health facilities were also significantly related to PNC use. Mothers who did not desire their pregnancy were more likely to use PNC at health institutions than their counterparts, which is reinforced by a study done in Ethiopia [49]. The consistency might be explained by fear of problems emerging after birth among mothers with undesired pregnancies. Likewise, mothers who had no big problem reaching a health institution were more likely to adhere to PNC utilization than those who had a big challenge to arrive at the health facility. This finding is contradicted with a study done in the Tigray region [50] which reported that the proximity of women to health institutions had an adverse relationship with the rate of PNC use. This could be clarified that individuals residing near to health institution might be developed poor health-seeking behavior. Moreover, mothers who are working their business were more likely to use PNC service than those who did not work their business which is supported by a study done in Uganda [51].

### Strength and limitation of the study

The strength of this study was the authors’ had been used nationally representative data from a consistent data set with the best response rates. Despite the findings of the study are precious for designing strategy and policy there are some critical barriers. As an example, the information composed is self-stated, which is liable for reporting errors and biases. Since it is a cross-sectional study, we could not assign causation to any of the relations among the known causes and the dependent variable. Nevertheless, this study is one of the few that has contributed in perspective of insurance enrolment and maternal health care service use among women in Ethiopia.

## Conclusions

This research showed that women enrolled in health insurance were associated with skilled delivery and recommended ANC utilization than women who did not enrolment in health insurance. Additionally, obstetrics and socio-demographic factors which include; age, education level, wealth index, marital status, place of residence, distance to a health facility, media access, occupation, parity, and pregnancy intention were substantial factors of maternal healthcare service utilization. So, improving health insurance coverage alone might not get the desired outcome in all maternal health services. It is also a critical issue to address the tackles of the entire health system to have an excellent maternal healthcare service in Ethiopia. The Ethiopian government should provide and strengthen health insurance enrolment for pregnant women especially those of lower socioeconomic status, uneducated women, those who had no access to media, those who were living far from health facilities, and women residing in rural areas. Healthcare providers should be strengthening health insurance coverage to enhance institutional delivery. Additionally, government and stakeholders’ efforts should be strengthened to stepping-up health insurance on uneducated and rural women in the lowest wealth quantile.

The findings of this study will also guide policymakers to develop an appropriate strategy and implementation plan about how good health insurance coverage affects the quality of maternal health services utilization.

### Recommendation for public health actions

The health care provider should provide knowledge about the significance of health insurance especially for those individuals who have low socioeconomic status and the rural residents.

Future researches should focus on assessing the usefulness of health insurance programs in endorsing maternal health service utilization among diverse socioeconomic populations using a strong research design.

## Data Availability

We recycled the USAID-DHS program 2016 Ethiopian demographic and health survey data set. To request the similar or altered data for another purpose, a new research project request should be succumbed to the DHS program here: https://dhsprogram.com/data/available-datasets.cfm The DHS Program will normally assess all data requests within 24–48 h (during working days) and provide notification if access has been approved, or other project information is needed before access can be granted. After getting consent, the investigator can log in and choose the specific data in the format they favor.

## Abbreviations

AOR: Adjusted Odds Ratio
ANC: Antenatal Care
CI: Confidence Interval
EDHS: Ethiopian Demographic Health Survey
EAs: Enumeration Areas
PHC: Population and Housing Census
MMR: Maternal Mortality Ratio
PNC: Postnatal Care
SPSS: Statistical Package Software for Social Science
UHC: Universal Health Coverage
WHO: World Health Organizations
